# Autonomous learning of pathologists’ cancer grading rules

**DOI:** 10.1101/2025.03.18.643999

**Published:** 2025-04-07

**Authors:** Thuong Nguyen, Vandana Panwar, Vipul Jamale, Averi Perny, Cecilia Dusek, Qi Cai, Payal Kapur, Gaudenz Danuser, Satwik Rajaram

**Affiliations:** 1 Lyda Hill Department of Bioinformatics, University of Texas Southwestern Medical Center, Dallas, TX, USA; 2 Department of Pathology, University of Texas Southwestern Medical Center, Dallas, TX, USA; 3 Kidney Cancer Program, Simmons Comprehensive Cancer Center, University of Texas Southwestern Medical Center, Dallas, TX, USA; 4 Department of Urology, University of Texas Southwestern Medical Center at Dallas, Dallas, TX, USA

## Abstract

Deep learning (DL) algorithms have demonstrated remarkable proficiency in histopathology classification tasks, presenting an opportunity to discover disease-related features escaping visual inspection. However, the "black box" nature of DL obfuscates the basis of the classification. Here, we develop an algorithm for interpretable Deep Learning (IDL) that sheds light on the links between tissue morphology and cancer biology. We make use of a generative model trained to represent images via a combination of a semantic latent space and a noise vector to capture low level image details. We traversed the latent space so as to induce prototypical image changes associated with the disease state, which we identified via a second DL model. Applied to a dataset of clear cell renal cell carcinoma (ccRCC) tissue images the AI system pinpoints nuclear size and nucleolus density in tumor cells (but not other cell types) as the decisive features of tumor progression from grade 1 to grade 4 – mirroring the rules that have been used for decades in the clinic and are taught in textbooks. Moreover, the AI system posits a decrease in vasculature with increasing grade. While the association has been illustrated by some previous reports, the correlation is not part of currently implemented grading systems. These results indicate the potential of IDL to autonomously formalize the connection between the histopathological presentation of a disease and the underlying tissue architectural drivers.

## INTRODUCTION

**Figure 1.**
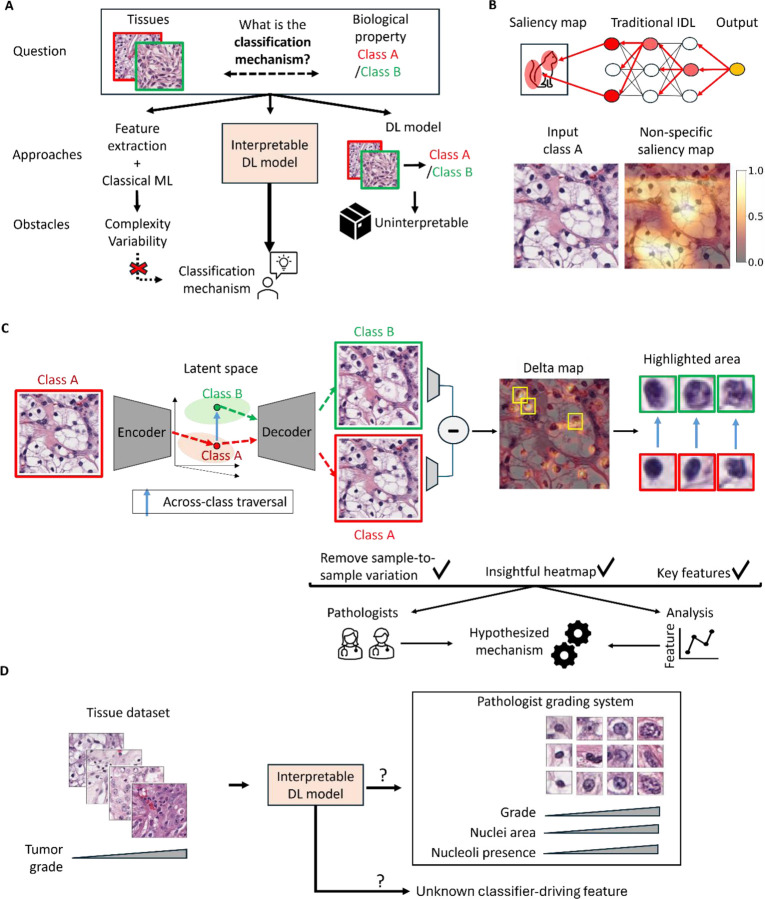
Overview of the study. **(A) U**nderstanding of the association between tissue morphology and its biology has evolved through centuries of observation and empirical study. In recent years, rule-building by human inspection has been complemented by classical machine learning (ML) techniques that can analyze features across biological categories by rigorous statistics. However, tissue complexity and within- and across-tumor variability hinders the engineering and interpretation of meaningful features. Deep learning (DL) excels in the classification of disease states by tapping into complex image features that are inaccessible to humans. However, DL models are seen as "black boxes" with limited transparency on which image information drives the classification. Efforts in developing interpretable DL (IDL) models have attempted to overcome this deficit by revealing the links between input data and predictions. **(B)** Traditional IDL methods tend to rely on saliency maps that mark classifier-informing regions in broad strokes but fail to extract from histopathological data the local and often spatially dispersed image properties that are critical for the classifier performance. **(C)** The IDL framework proposed in this work implements an autoencoder model and latent space traversal (blue arrow) to generate synthetic images of the same tissue with different classes. These synthetic images are utilized to create a Delta-Map, which underscores significant across-class transformations and provides insights into the mechanisms driving morphological changes associated with biological progression. **(D)** Definition of goals of the present study: Evaluate the ability of the proposed IDL model to autonomously learn clinically-established tumor grading rules and in potentially creating new rules that may inform an enhanced tumor grading procedure.

Pathologists’ assessment of tissue morphology to determine cancer subtype, aggressiveness and progression^1–10^ plays a critical role in oncological clinical decision making^11–15^. The rules underlying these assessments have evolved over decades of detailed observation to empirically balance predictive power with reproducibility across observers. There have been many efforts to discover novel rules by computationally scaling up the process of extracting morphological features and training “classical” machine learning (ML) models (Fig. 1A). In practice, the ability to extract the “correct” features has been a bottleneck^16^. Traits commonly used by pathologists (e.g., cell type) are non-trivial to engineer, while novel discriminative features in cancers are often unintuitive due to tissue complexity and tumor variability, making them hard to formulate^17–28^. In contrast, deep learning (DL) algorithms, which bypass the task of feature engineering, have excelled on a wide range^9, 29–33^ of histopathology-based predictions, matching human performance on traditional classification tasks and enabled predictions of mutation status, gene expression, molecular subtypes and treatment response^34–36^. However, the "black box" nature of DL models precludes the identification of specific morphological biomarkers representing the specific disease-driving processes. Traditional interpretable deep learning (IDL) methods^37–45^ for image data are based on identifying the regions that most influence the model’s classification (Fig. 1B). Unfortunately, the disease-relevant patterns of tissue morphology are not localized like those in natural images, and the large swaths of the image that tend to be highlighted by the IDL offer little insight beyond the types of cells present (Fig. 1B, S1). There is thus a pressing need for IDL methods that are better suited to tissue morphology^37–50^.

We previously developed a conceptually distinct IDL approach leveraging generative artificial intelligence (AI) to explore the morphological basis of an image-based classifier of metastatic propensity of single melanoma cells in culture^51^. Specifically, we trained a latent representation capable of generating images of cells with different levels of metastatic spreading. By examining the warping of images as we traversed the latent space between areas of high and low metastatic propensity, we identified extension of pseudopods as the defining feature of high metastatic potential. Both the classifier and the IDL-predicted morphological behavior of aggressively metastasizing melanoma cells were validated in follow-up studies using human xenografts in mice^51, 52^. Of note, it would be very difficult to human-engineer a direct detector of pseudopodal extension in these images, and the cell-to-cell heterogeneity prevented us from visually noticing the differential in pseudopodia in the experimental dataset. Thus, our discovery was made possible by the power of generative AI to gradually parse the complex mix of phenotypes across cells with differing metastatic potential even beyond the range of available experimental data. While several studies^53–55^ have since applied generative AI models to histopathology for expanding datasets and visualizing class transformations^56–64^, the potential of generative AI and as an IDL approach to elucidate the relationship between tissue structure and disease state has remained largely untapped.

Here we design an IDL framework (Fig. 1C) that extends the concept of interpretability via latent space traversal from single cells to patient tissue samples via two key innovations. First, we adopt diffusion models (DMs)^65–70^ to learn a meaningful high level semantic latent space that is agnostic to interchangeable low level image features, which are nonetheless crucial for the production of realistic images (Fig. 1C). Second, we introduce the concept of the *Delta-March* to analyze changes between counterfactual images – images of the same tissue that differ in class – produced by the latent space traversal and identify the disease driving features. As a test case, we demonstrate that the proposed IDL model faithfully recovers the clinically applied grading rules for clear cell renal cell carcinoma (ccRCC). The IDL model also defines an additional rule that, while accepted by pathologists as plausible, has yet to be entered into the official grading scheme. This provides a glimpse of the potential of this AI framework to complement existing diagnostic approaches with novel biomarkers and to assist clinicians in the generation of hypotheses on cancer-driving biology that has so far escaped the current body of medical knowledge.

## RESULTS

### Testing the IDL model in capturing tumor grading rule

Pathologists classify ccRCC into four grades of tumor aggressiveness based on hematoxylin and eosin (H&E) stained histopathology slides. The scoring systems have been updated over time but are largely focused on nuclear size and nucleolar prominence, both of which increase with higher-grade aggressive tumors^71–73^. Specifically, distinctions of grades 1, 2, and 3 are purely based on these features, while grade 4 also considers non-nuclear properties of tumor cells such as sarcomatous or rhabdoid differentiation, which are associated with poor prognosis and more aggressive tumor behavior^71–73^. Additionally, while not part of the official grading rules, the extent of vasculature is known to decrease with increasing grade^74–76^. We note that ccRCC tumors exhibit a high degree of intra-tumor heterogeneity^29^, and grade is traditionally assigned at a patient level based on the highest grade area within a tumor. We employ a dataset of 1,128 whole-slide images from about 1,120 patients (Methods), with patch level tumor grade assignments rather than conventional patient-level grading, to test the ability of the proposed IDL approach to autonomously recover the same or alternative grading criteria (Fig. 1D).

### Unsupervised Autoencoder

**Figure 2.**
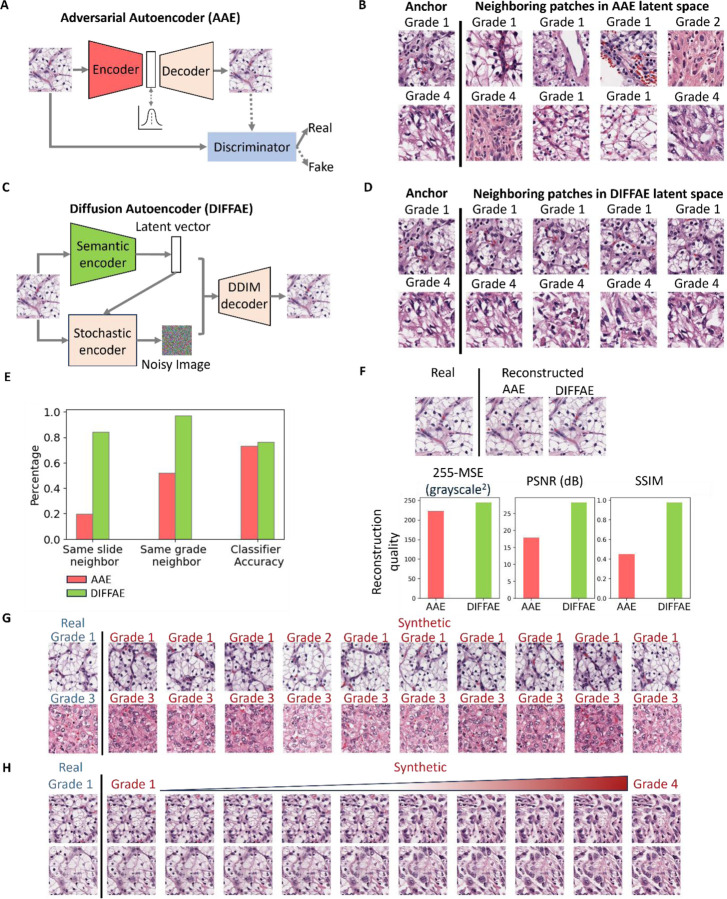
Unsupervised Autoencoder models for IDL framework. **(A)** Structure of Adversarial Autoencoder (AAE) model for tissue images, comprising a 1024-dimensional latent space and a discriminator model to distinguish between original and reconstructed images, thereby encouraging the AAE model to produce images that are indistinguishable from the originals. **(B)** Two examples (rows) of patches and their nearest neighbors in the 1024-dimensional AAE latent space. These patches exhibit differing morphology and tumor grades, indicating that the AAE latent space is discontinuous in these variables. **(C)** Structure of Diffusion Autoencoder (DIFFAE) comprising a 512-dimensional semantic encoder and a stochastic encoder. **(D)** Examples of neighboring patches in latent space obtained by DIFFAE exhibit consistent morphological and tumor grade characteristics, indicating the increased continuity in these variables. **(E) C**omparison of the percentage of 10 nearest patches (N=120,000) of a patch which come from the same slides or have the same grade based on the AAE (pink) and DIFFAE (green) latent spaces. Accuracy of an LDA classifier to distinguish between low and high-grade patches in the respective latent spaces. **(F)** Comparison of images reconstructed by AAE and DIFFAE. Accuracy of image reconstruction in AAE and DIFFAE (N=1000 patches), measured using MSE, PSNR, and SSIM. **(G)** Demonstration of the interaction between semantic latent vector and noise image of the stochastic encoder. Two tissue images (rows) were encoded into semantic vectors, combined with 10 random noise inputs to synthesize images. Synthetic images on same row share the input tissue phenotype and (with the exception of 1 patch) have the same grade as predicted by VGG19 tumor grade classifier but differ in spatial structure. Images in the same column share the spatial patterns as they are generated by the same noise inputs. **(H)** Demonstration of tumor grade transformation by traversal of the semantic latent space while preserving spatial structure. Semantic vectors and stochastic noise images are extracted from two grade 1 tissue image patches. The semantic vectors were then shifted along the linear classifier axis toward grade 4 and combined with the unchanged noise images in the DIFFAE decoder to generate a synthetic image sequence illustrating a smooth transition of tissue characteristics across grades.

We first sought to generate latent space representations of H&E patches via the Adversarial Autoencoder (AAE) model previously used for melanoma cell classification^51^. Briefly, this approach employed an autoencoder to represent the image by a 56-dimensional latent feature vector, from which it is possible to reconstruct the full input image. To adapt this approach to the structurally more complex tissue image patches, we increased the latent space dimension and added an adversarial arm to encourage the reconstructed images to be indistinguishable from the real ones (Fig. 2A). Although the AAE produced visually accurate reconstructions (Fig. S2A), the underlying latent space was biologically sub-optimal: image patches with highly similar latent vectors often shared a similar tissue architecture, but could exhibit highly different traits, such as originating from different slides and grades (Fig. 2B, S2B). This phenomenon was true regardless of the latent vector size we chose (Fig. S2B). We reasoned that the discordant nature of the AAE's latent space arises from the representations having to perfectly recover all aspects of the image, including many trivial aspects such as absolute positions of nuclei that are unrelated to the tumor grade.

To overcome the need for specifying low level detail in our latent vector, we adopted diffusion models that are widely used^65^ for generating high quality images based on broad descriptive prompts (e.g., DALL-E^65^). Diffusion models^66, 67, 69, 70^ learn to generate the full spectrum of training images by iteratively denoising a random signal, and the prompt vector associated with each image merely serves as a bias directing this process. Specifically, we chose to replace the AAE with a Diffusion Autoencoder^66^ where the image representation is effectively bifurcated into two parts^66^ (Fig. 2C): the latent vector captures the high-level, semantic image content, whereas the low level interchangeable image aspects (such as the absolute locations of nuclei) are delegated to the stochastic encoder. Compared to the AAE, DIFFAE i) generates a more biologically meaningful latent space in which tissue images with similar latent vectors originate from the same slide and grades (Fig. 2D, E); ii) provides better separability of grades based on Linear Discriminant Analysis (LDA) in the semantic latent space (Fig. 2E); and ultimately iii) also improves the quality of image reconstructions, as assessed by visual inspection and standard image quality metrics (Fig. 2F, S2C). Together, these advantages allow the DIFFAE to generate different tissue images representing the same grade (Fig. 2G) and to simulate the different appearances of the same tissue as it would progress from Grade 1 to Grade 4 (Fig. 2H). The former image series are generated by varying the noise component for a fixed position in the semantic latent space. The latter image series are generated by traversing the sematic latent space (Methods) from regions encapsulating Grade 1 to regions encapsulating Grade 4, leaving the noise component fixed. The direction of traversal in this case is defined by the vector of maximal grade separation as determined by a 4-class linear classifier.

### Evaluation of synthetic tumor grade data by human pathologists

**Figure 3:**
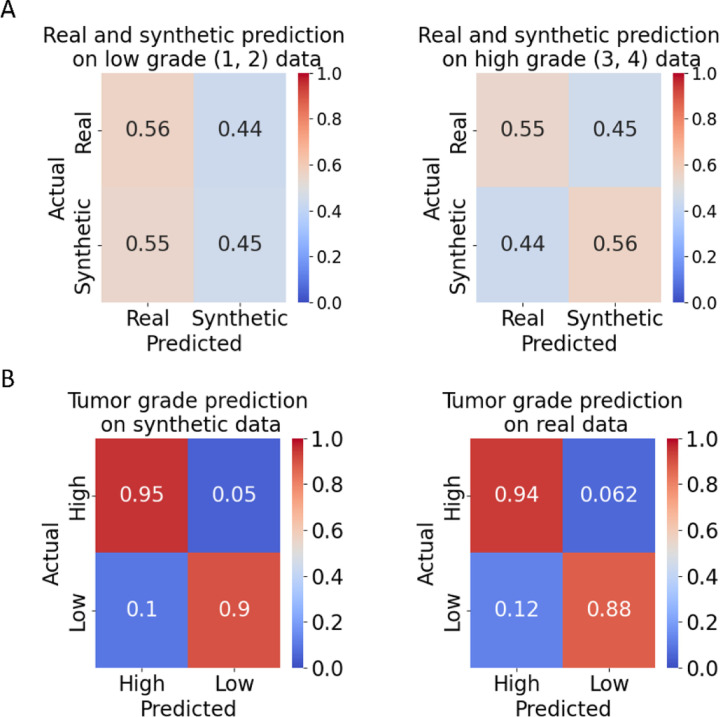
Classification of DIFFAE-generated images by expert pathologists. Four pathologists were tasked with determining for N=80 tissue image patches whether i) the image was real or synthetic and ii) it displays a low grade (grades 1 or 2) or high grade (grades 3 or 4) tumor. **(A)** Accuracy of distinguishing real from synthetic images at low (left) or high (right) grade. The average accuracy obtained is comparable to a random chance level regardless of tumor grade. **(B)** Tumor grading accuracy on synthetic (left) and real (right) data. Ground truth grade levels were determined by grade classification model (see Fig. S3 for between pathologist agreement).

We sought to gauge the realism of the DIFFAE-synthesized images by engaging the inspection of experienced pathologists who were blinded for the source ‘synthetic’ vs ‘real’ and grade of the tumor tissue image patch. Specifically, using the DIFFAE we generated 40 synthetic images equally split across all four grades (as determined by our grade model) and merged them with a similarly distributed set of 40 real images. We then presented these 80 images one–by-one in randomized order to four pathologists with experience in ccRCC pathology. They were tasked with specifying for each patch the source (real vs synthetic) and grade (low: 1/2 or high: 3/4). The ability to distinguish real from synthetic images was no better than random (45% vs 56%) for both low- and high-grade images (Fig. 3A). On average pathologists achieved 92% accuracy in identifying the grade on both real and synthetic images (Fig. 3B) with ground truth based on our grade model and similar results using individual pathologists to assign grade (Fig S3A). Albeit not statistically significant, lower accuracies were observed in classifying low-grade images both real and synthetic, with about 11% being mistaken for high-grade. This possibly reflects the challenge of grading small patches: as grade calls are based on the most aggressive cells, a small number of cells with more aggressive appearing phenotypes could lead to a high-grade call in a low-grade region. The tumor grading performance was comparable on real and synthetic data both in terms of agreement with the grade model (Fig S3B, C) and among pathologists (Fig. S3A). Overall, we conclude that the DIFFAE model generates realistic tissue images that preserve the characteristics of assigned tumor grades.

### Leveraging synthetic image series of grade transitions to define grade-driving image features

**Figure 4.**
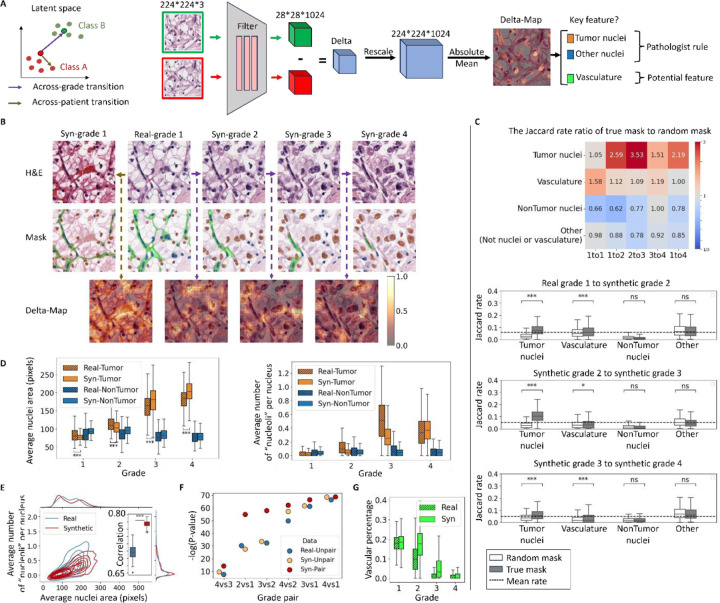
The capacity of the IDL model to generate hypotheses on grade-driving mechanisms by Delta-Marches. **(A)** Overview of the Delta-March framework to identify image features that vary across image classes. Rather than working directly in the image space, the proposed IDL relies on the detection of spatially distributed features that significantly alter during a semantic latent space traversal in the direction of grade transitions. Within class (across patient) transitions serve as the negative control. **(B)** An example of the Delta-March. Depicting the real/synthetic images from the latent space transition (top) the corresponding cell types (middle) and an overlay of the Delta-Map intensities between successive steps (bottom) during latent space transitions between indicated tumor grades. **(C)** Jaccard similarity analysis comparing Delta-Maps to tissue segmentations. For N=977 real grade 1 samples transitioning through four grades, Delta-Maps were binarized (threshold = 0.5) and evaluated, against both actual tissue segmentations and random masks, using the Jaccard index (intersection over union). The table displays the median ratio of Jaccard indexes for actual masks to random masks, revealing the prominent variation of tumor nuclei during grade transitions. Negative controls, based on within-grade (across-patient) transitions, exhibit distinct patterns, supporting the specificity of tumor nucleus variation in across-grade transitions. Bar plots depict Jaccard indexes for indicated tissue components during grade transitions. Dash line is the average Jaccard rate across all samples and components in each transition. **(D)** Variation of tumor and non-tumor nuclear phenotypes across grades in real and synthetic data (N=500 patches per grade). Synthetic grades 1–2 patches are generated by traversing from real grade 4 images (N = 556) toward grade 1, and grades 3–4 patches from grade 1 (N = 977) toward grade 4. Both datasets show consistent trends of increased tumor nucleus size and nucleoli count with higher grades, while non-tumor nuclei do not exhibit these trends. Nucleoli are identified as local extrema in the hematoxylin channel, considering them as “nucleoli”. **(E)** Relationship between tumor nuclear area and “nucleoli” number in real and synthetic data. Contour plot highlights the joint distribution (N=500). Inset, correlation between nuclear size and nucleolar count (with N=100 random samples of 100 patches per grade) in real vs synthetic data **(F)** Comparison of the statistical power to detect increase in per-patch average tumor nuclear size with grade using samples from real and synthetic data. In the real data, for each grade pair comparison, images (N=100 for each grade) were randomly sampled (Real-Unpair), while in the synthetic data, images were generated either from the same (Syn-Pair) or from independent (Syn-Unpair) latent transitions from grade 1 to grade 4. The sign test was used to compare average nuclear size between two groups (N = 100 patches), repeated 1,000 times and to compute the mean of log(p-value) across repetitions. **(G)** Analysis of percentage area covered by vasculature across grade. The coverage decreases in both real and synthetic data (N=500 patches per grade).

Our goal for the IDL system is to autonomously identify image features that distinguish specific sample properties of interest – here, cancer grades – and aid pathologists in formulating hypotheses on the underlying property-driving mechanisms. To this end, we developed an analysis referred to as *Delta-March* (Fig. 4A) that facilitates the identification of phenotypes linked to changes in the specific sample properties. Rather than comparing different tissue images across different grades, which vary in numerous other aspects besides the grade, Delta-Marches reveal how a particular tissue patch changes solely because of a grade transformation. To nominate the most decisive grade-driving features in complex tissue images, we utilize the encoder in the pretrained AAE as a means of extracting lower-dimensional, abstract image representations and subsequently track hotspots of representation changes, captured in a *Delta-Map*, between consecutive steps of the Delta-March.

The Delta-Maps across grade transitions demonstrate a high degree of spatial modulation with localized attention hotspots (Fig. 4B), significantly surpassing typical class activation mapping techniques Fig. S1. Intriguingly, visual inspection of these results highlights a focus on tumor nuclei, consistent with grading rules. The significance of these hotspots can be appreciated in comparison to a direct jump from grade 1 to 4 (Fig. S4) and to a within-grade negative control (Fig. 4B left column), where we preserve the noisy image (and hence the position of nuclei) of the initial patch but replace its semantic latent vector with that of a different patch of the same grade. For a more systematic and unbiased analysis, we cross-referenced the Delta-Maps with segmentations of tumor and other nuclei, as well as the vascular network^76^. We then evaluated the enrichment of the Delta-Maps intensity across these segmented areas. Specifically, we binarized the Delta-Maps into low- and high-intensity regions and calculated the Jaccard similarity index, defined as the ratio of the intersection to the union between the binarized Delta-Map and the segmentation mask. Additionally, we compared this Jaccard index to a random baseline, calculated using segmentation masks from a different randomly selected patch of the same grade. The table in Fig. 4C presents the ratio of Jaccard indexes (actual mask to random mask). Higher ratios indicate stronger alignment of the Delta-Map with the segmented tissue component of interest. The ratios reveal that the Delta-Map produced by marches across grade transitions is significantly elevated at the loci of tumor nuclei, but not at the loci of non-tumor nuclei (e.g. immune cells and other cell types) (Fig. 4C). Hence, the IDL determines that cancer cell nuclei are a key indicator of grade transitions, as postulated by today’s clinically implemented grading rules^71–73^. Interestingly, the decreased focus on tumor nuclei between grades 3 and 4 (bar plots Fig. 4C) is consistent with the inclusion of non-nuclear tumor cell phenotypes in calling grade 4 (as opposed to grades 1–3 which are purely nuclear). Negative controls, based on within-grade (across-patient) transitions, show lower and less specific ratios, further demonstrating the unique roles tumor nuclei ought to play in across-grade transitions. Although the Jaccard indexes were relatively high in the other regions, i.e. tissue parts not associated with cell nuclei or vasculature derived from true masks, they were lower than those obtained from the random masks leading to ratios below 1. This suggests that no major features exist in the tissue background that would determine the grade transition. Finally, while the Jaccard indexes associated with vasculature segmentation across grade transitions were lower than those for tumor nuclei, they were still higher than the indexes from random masks, i.e. index the ratios are consistently above 1 for all grade transitions. This indicates that vasculature may be a secondary marker of tumor grade. Interestingly, small-step marches captured vascular changes, whereas direct large jumps from grade 1 to grade 4 overlooked these subtle features. This underscores the advantages of Delta-March, which integrates changes in tissue appearance over a continuous, controlled progression, allowing the detection of subtle grade-related morphological shifts that may be obscured by tissue heterogeneity and variation agnostic to the grade transition.

Next, we focused on the putative phenotypic changes in nuclei from across-grade transitions in synthetic tissue image patches (Fig. 4D). We found that tumor cell nuclei, but not nuclei of other cell types, increased in size with grade, highlighting the cell-type awareness of the semantic latent space. Moreover, the count of peaks of hematoxylin intensity per nucleus, which we use as a proxy for prominent “nucleoli” (Methods), also grew only in tumor cells (Fig. 4D). Notably, the change in nuclear size and nucleoli between grade 3 and grade 4 was relatively less pronounced compared to other grade transitions in both real and synthetic data, reflecting nuclear features are not the primary distinguishing feature for grade 4 classification. This shows that the IDL system based on Delta-Marches reproduces established grading rules directly for the data. Interestingly, the tumor grade effect on nuclei is more pronounced in grade transitions in the semantic latent space, with significantly smaller nuclei in synthetic low grades (1–2) and larger nuclei in synthetic high grades (3–4) compared to real data (Fig. 4D). While the individual trends for nuclei and “nucleoli” are largely in agreement between real and synthetic data, the correlation between these two features is stronger in the synthetic data (Fig. 4E, S5). Thus, while real image patches display some of the expected grade related features, these are more consistently present in synthetic data.

We sought to more formally test whether the ability of latent space traversals to solely modulate grade increased the statistical power over traditional cross-grade comparison where other non-grade aspects may also change. Specifically, we wondered to what extent any differences arose from our synthetic trajectories exaggerating grade features as opposed to overcoming interpatient heterogeneity within a grade by following the same patch across grades. To address this, we compared the average nuclear size across tumor grades (1 to 4) using both real and synthetic data. In the real data, images were randomly sampled from each grade group (Real-Unpair). For synthetic data, two approaches were used for each grade-pair comparison: (1) Syn-Unpair, where samples originated from independent latent space transitions, which would allow us to test solely the difference between synthetic and real feature distributions (2) Syn-Pair, where counterfactual samples were derived from the same latent space trajectories grade 1 to grade 4, thereby potentially overcoming interpatch variability. For each grade pair, we applied the sign test to compare the per-patch average nuclear size between groups (N = 100 patches), repeating the analysis 1,000 times. The modest and inconsistent p-value improvements in Syn-Unpair (Fig. 4F) suggest that feature amplification alone has a limited impact on statistical separation. In contrast, the consistently smaller p-values observed in the Syn-Pair data highlight the IDL system’s ability – through counterfactual analysis – to emphasize grade-relevant tissue features more effectively than conventional cross-grade comparisons. Notably, the synthetic pair comparisons produced the most significant results, with p-values up to 10^20^ times smaller than those observed in real data for adjacent grade pairs (Fig. 4F), where strong correlations within a trajectory help control for heterogeneity. For more distant grades, the correlation between nuclear sizes weakens, and differences are increasingly driven by distributional shifts. While in this case, the nuclear size has a strong enough effect to be detected also in real data, this approach will allow us to pinpoint more complex traits with smaller effect sizes and in more data-limited applications. Taken together these findings highlight two key advantages of the proposed Delta-March: (1) the ability to isolate the effect of individual variables on phenotype changes using analysis of counterfactual images, and (2) the use of small, controlled transitions along a biologically meaningful latent axis to enhance statistical power and provide a more precise and interpretable means of uncovering underlying disease mechanisms.

Finally, we were curious to see whether the reduction in vasculature with grade previously conjectured by us and others^76^ was also reflected in synthetic image series of latent space transitions in the direction from low to high tumor grades. We found indeed that the model recapitulated this effect to a level comparable to that in real data (Fig. 4G). We note that unlike tumor-nuclei, within grade transitions seemed to impact the vasculature pattern (Fig. 4C, 1st column), possibly because there are non-grade related aspects that also impact the specific vascular pattern observed.

## DISCUSSION

There are three fundamental challenges in using real world data to identify meaningful rules connecting tissue morphology to disease properties of interest (e.g. mutation status of a single gene or tumor aggressiveness for which grade is a proxy). First, morphological traits manifest highly non-linearly as image features, and much of the variation in the image pixel space, such as absolute positions of nuclei, is irrelevant. Second, it is rare to find samples differing solely in the disease property of interest, and thus observed morphologic changes across specimens are confounded by differences in non-related properties. Third, even if we could solely change the disease property of interest, large biological changes (e.g. a jump from grade 1 to 4) will impact multifarious aspects of morphology due to the complex inter-connected networks of influence. In attempting to overcome these challenges, we take inspiration from the production of caricatures where cartoonists identify the essence of a subject they wish to highlight and then depict it with a minimal set of strokes. Translating this process into an AI system, we address these challenges by a) using a DIFFAE to produce meaningful latent representations of images that consigns irrelevant parts into a noise vector, b) identifying a direction in the latent space such that changes are solely associated with the property of interest, and by c) traversing the latent space in a Delta-March along this direction to identify the most salient affected regions in the image. Thus, the presented IDL framework serves as an AI-powered cartoonist that, when presented with representative data of both consequential and inconsequential variations, autonomously extracts the association between tissue appearance and the property of interest.

While the proposed strategy is agnostic to data type and biological process, we provide proof of principle demonstration in learning grading rules for ccRCC pathology. Our previous IDL approach for single cell images^51^ used a simpler AAE encoder and needed to exaggerate effects beyond the naturally observed variation to highlight pseudopodal extensions as a readout of metastatic propensity. Here in a more complex tissue setting, we used a more sophisticated approach to removing distracting features and highlight features of interest. First, we showed that the DIFFAE’s split of the latent space into meaningful and “noise” aspects (associated e.g., with nuclear positions) results in a more biologically meaningful representation of histopathology images than a simple AAE. Next, by moving in the direction of grade change in the latent space we synthetically warped images to simulate tumor grade transitions while holding other aspects of tumor tissue variation fixed. The resulting synthetic images more consistently exhibited changes to multiple grade-related image features (nuclear size and nucleolar count) than real images (Fig. 4D, E) and even led to a slight (albeit not significant) increase in pathologists’ classification of grades (Fig. 3B). Moreover, comparing nuclei to their synthetically grade warped versions increased statistical power relative to real samples differing in grade (Fig. 4F), suggesting our counterfactual approach to overcome sample to sample variation. This could enable detection of subtle effects for which available experimental data do not provide adequate power. Finally, the Delta-March enables a continuous, controlled simulation of the increase of grade on individual cells with high spatial resolution, thereby allowing us to identify a minimal set of grade-associated changes. Specifically, our Delta-March process highlighted the tumor nuclei as most impacted, allowing us to then recover the known grading rule of increased nuclear size while the increase in number of local intnsity maxima within the nuclear masks mirrors the rule of increased nucleolar prominence. Interestingly, in identifying the decrease of vasculature with grades which we and others have noted before^74–76^, our approach goes beyond the formally identified grading rules. VHL loss, an initiating event in ccRCC, causes increased vasculature as cells perceive themselves to be in hypoxic conditions^77^, but with increasing grade cells can survive without such vasculature, likely due to a metabolic shift away from oxidative metabolism towards the Warburg effect^78^. Taking together these results suggest our approach has the capacity to distill the essence of image properties associated with tumor grade change.

Future work can build on our work in several ways. Conditional diffusion models^67, 79^ have shown promise in transforming histopathologic images to reflect changes in molecular state, but the large image changes produced in these transformations limit interpretability. Our Delta-March type procedure instead uses smaller steps in a latent space to capture an integral of more subtle changes. More work is needed to determine the optimal nature of this space (e.g. learnt during the diffusion model training as done here or using pre-existing representations from foundation models^68, 80–82^), and its best traversal. If successful, such an approach has the ability to nominate novel relationships between morphology and genetic/disease state. Cancer is an excellent application area for the proposed IDL system because of the multifactorial root causes of disease progression and their manifestation in complex morphological signals. Other diseases with similar traits include neurodegeneration, where different strains (protein assemblies differing in structure) have been hypothesized to underlie complex aggregate morphologies that are the gold standard for disease classification^83^. More broadly, image classification and generation have traditionally been separate domains. Yet, as the adoption of machine learning algorithms in biomedical domains is limited by their interpretability rather than their classification performance, we anticipate that the development of representations that enable both classification and generative visualization of this space will become important. Thus, our study paves the way for deeper investigations into disease mechanisms, providing a tool to explore how morphological features may correlate in unexpected ways with disease progression and treatment outcomes. We anticipate that the proposed IDL system will uncover patterns in tissue samples that will inform both clinicians and life scientists in their quests to deliver more specific and personalized treatment, and to identify the processes that govern a particular pathophysiology.

## MATERIALS AND METHODS

### Data

All analysis makes use of 224x224-pixel image patches at 20X magnification (~0.5 microns per pixel) extracted from “tumor” regions of Hematoxylin and Eosin (H&E)-stained whole slide images of clear cell renal cell carcinoma (ccRCC). The image patches are drawn from one of two cohorts:

1. Grade cohort: Consists of 128 slides within which a pathologist (P. Panwar) annotated tumor regions and assigned them specific grades (1–4), as opposed to using a single patient level grade as is traditional in the clinic. Patches were randomly drawn from these annotations and inherited the grade of their parent annotation.
2. Mayo Cohort: 1,000 WSI from Mayo Clinic repository which we previously published^84^. A pretrained region classification model was used to identify tumor regions in these slides, and patches were drawn randomly within these regions.

### Adversarial Autoencoder (AAE)

#### Architecture:

The Adversarial Autoencoder (AAE) model adopts an unsupervised framework inspired by^51^, employing a convolutional encoder-decoder architecture. The encoder compresses input data through five convolutional blocks, incrementally increasing channel sizes (256, 512, 1024, 1024, and 1024), each paired with batch normalization and Leaky ReLU activation layers. Latent space representations are generated via a fully connected layer. The decoder reverses this process, reconstructing the original input via transposed convolutional layers, batch normalization, and sigmoid activations. Following the original AAE paper^85^, our model uses an adversarial component intended to make the latent distribution indistinguishable from a normal distribution. Additionally, to enhance the reconstruction quality of high-resolution tissue images, we added another adversarial component, similar to those used in Generative Adversarial Networks (GANs), that ensure the real and synthetic images were indistinguishable. This discriminator leverages five convolutional layers with batch normalization and Leaky ReLU activations, concluding with a fully connected layer and sigmoid output.

#### Training:

As this model is an autoencoder the training is completely unsupervised. However, to ensure that the model sees a wide distribution of grades, for training and testing, we utilized 120,000 patches, with 30,000 patches per tumor grade derived from the Grade cohorts. Training involved minimizing a composite loss function comprising reconstruction, adversarial, and discriminator loss terms, balanced for optimal performance. The model was trained for 30 epochs with a fixed learning rate *r* = 10^−4^ for the AAE and *r* = 10^−5^ for the discriminator components, respectively.

### Diffusion Autoencoder (DIFFAE)

#### Architecture:

We adopted the DIFFAE model^66^ from https://github.com/phizaz/diffae. Briefly, the architecture of a diffusion autoencoder integrates an encoder, a diffusion process, and a decoder to create a robust generative framework. The encoder compresses input data into a latent representation, which is then progressively corrupted through a series of controlled noise-injecting steps in the forward diffusion process. The decoder reverses these steps, refining the latent representation to reconstruct the original input. For our experiments, we retained all default parameter values from the original implementation, with adjustments made solely for the input image dimensions (224x224 pixels).

#### Training:

For comparative analysis (Fig. 2) with AAE model’s performance, we trained the DIFFAE model on a subset of 8,000 patches taken from the 120,000 patches used in training for AAE model evenly distributed across tumor grades. Next, to enhance model robustness and accommodate tissue structural variability, we subsequently trained the unsupervised DIFFAE model on an extended dataset of 18,000 images curated by adding 10,000 unannotated tissue patches extracted from the Mayo Cohort.

### Image Grade Classification and CAM

We trained a grade classifier with VGG19 backbone on 120,000 patches over 50 epochs with a learning rate of 10^−4^, achieving 92% accuracy on the training dataset. The VGG19 model was pretrained on ImageNet dataset and fine-tuned for four-grade classification using cross-entropy loss.

Class activation map (CAM) is a widely used technique for generating heatmaps that visualize important regions influencing CNN and deep learning model predictions. To validate the performance of CAM-based approaches on detecting critical features related to tumor grade, we performed GradCam++, XGradCAM, and AblationCAM for our pretrained VGG19 grade classifier focusing on the last convolution layer. These CAM-based models are taken from pytorch_grad_cam package. The obtained heatmaps (Fig. S1) highlight nuclear regions but exhibit diffuse patterns, lacking precise localization of critical features for tumor grading, limiting their interpretability.

### DIFFAE latent space transitions

Our methodology for class transitions is based on the attribute manipulation technique from the DIFFAE framework^66^. Specifically, in addition to the unsupervised DIFFAE framework, ^66^ introduces a logistic regression classifier trained on latent vectors from the semantic encoder to identify the axes that separates classes. Attribute manipulation is then performed by traversing the semantic vector along the corresponding axis. In our work, we applied this approach to 8,000 annotated patches across four tumor grades, analyzing the projection of latent spaces onto classifier axes (Fig. S6). The weight vector for grade 4 shows a continuous distribution spanning grades 1 to 4, with grades 2 and 3 positioned in between, while the grade 1 vector exhibits an inverse pattern. Therefore, to transition towards higher grades, we adjust the latent representation linearly along the direction of the grade 4 weight vector, while to transition towards lower grades, we shift it along the grade 1 weight vector

Namely, to traverse a real tissue sample from grade 1 to higher grades, we use the semantic and stochastic encoders of DIFFAE to obtain the tissue image's semantic vector and noisy image. The semantic vector is then standardized and incrementally shifted along the grade 4 weight vector in step sizes proportional to the square root of the vector’s dimensionality. This step size is used in ^66^ for isotropic random walks in 512-dimensional Euclidean space, ensures smooth progression between grades while maintaining the meaningful structure of the tissue representation. The shifted vector is subsequently de-standardized and passed to the DIFFAE decoder along with the original stochastic noisy image to generate synthetic tissue images. The transition halts either when the generated image is classified as grade 4 by the pretrained VGG19 tumor-grade classifier or after a maximum of four steps. Upon determining the endpoint, 20 intermediate semantic nodes are interpolated along the transition path. These nodes, along with the original noisy image, are input to the DIFFAE decoder to generate synthetic images depicting the across-grade transition. The tumor grades of these generated images are validated using the VGG19 classifier.

### Synthetic Data Sets:

Synthetic images for pathologist evaluation in Fig. 3 were generated through latent transitions starting from real samples of all four grades. Transitions toward grade 1 and grade 4 produced low and high grade representations, respectively; see Table S1 for details.

To learn the tumor grade, demonstrated in Fig. 4, we traversed 5,000 real grade 1 samples-assigned by both model prediction-toward grade 4. Of these, 977 transitioned across all four grades (1–4) with the highest-confidence images selected for each grade. For within-grade transitions (across patients), the noisy image of a tissue sample remains unchanged, while its semantic vector is replaced with that of a random tissue sample of the same grade from a different patient. To generate low grade tissues for Fig. 4D, E, and G, we reversed the process by shifting 5,000 real grade 4 patches toward grade 1, yielding 556 synthetic samples spanning all grades.

Additional visualizations of latent transitions are shown in Figures S4 and S7.

### Deep Learning filter for Delta-March

To identify phenotypes linked to class changes, we developed the Delta-March analysis pipeline (Fig. 4A). This pipeline uses a CNN filter derived from the first 9 layers of our pretrained AAE. Each image pair is passed through this filter, generating 28x28 feature arrays with 1024 channels. These feature arrays are subtracted to compute Delta arrays, which are then upscaled to match the original image dimensions of 224x224. The absolute values of the resulting arrays are averaged across all 1024 channels to produce Delta maps, highlighting areas of morphological difference between the two input images. Additionally, we also consider the Delta-Maps derived from the first 13 layers of our pretrained VGG19-based tumor grade classifier following a similar pipeline (Fig. S4, S7, and S8). We selected this depth for Delta-march filter to capture feature maps that retain low-level structural details while beginning to incorporate high-level tumor-grade features.

### Delta-March Transitions

We initially focused on the 977 across-grade transitions (described above) from grade 1 to grade 4, which pass through intermediate grades 2 and 3. To capture fine-grained morphological changes, the transitions were subdivided into sub-transitions: real grade 1 to synthetic grade 2, synthetic grade 2 to synthetic grade 3, and synthetic grade 3 to synthetic grade 4. We also extended the Delta-March analysis to across-patient transitions by swapping semantic vectors of tissue samples between different patients of the same grade, enabling a deeper understanding of tumor grading features.

### Delta-March average intensity rate

Next, we calculate the average intensity of the Delta-March across different tissue components: tumor nuclei, non-tumor nuclei, vascular vessels, and background. This analysis provided insights into key features influencing tumor grading. To execute this calculation, segmentation masks were generated for each sub-transition by merging the masks of the initial and final tissue images. Nuclear classification and vasculature segmentation are derived using two distinct segmentation models developed by the Rajaram lab^76^. The nuclear segmentation model classifies pixels into three categories: 2 = tumor nucleus, 1 = non-tumor nucleus, and 0 = non-nuclear. The vasculature segmentation model assigns pixels as either 1 = vasculature or 0 = non-vasculature. First, the segmentation masks from the initial and final tissue images were combined by assigning each pixel the maximum label present across both images. Then, the nuclear and vasculature segmentations were integrated into a single composite map. To prevent bias, pixels classified as both vascular and nuclear were randomly reassigned to either vasculature or their respective nuclear class. (tumor vs non-tumor). Additionally, we observed that some nuclei appear in one mask but not the other—an artifact commonly produced by generative models with no biological relevance. The Delta-March can detect these "ghost nuclei," potentially leading to misinterpretations of the underlying biological mechanisms. To mitigate this, we eliminate these "ghost nuclei" from the masks.

To evaluate which cell types showed morphological changes, we calculated the Jaccard similarity index to measure the intersection over union (IoU) between binarized Delta-maps and the segmentation masks as follows. First, the Delta-map values are normalized using the per-patch minimum. We then binarize the normalized arrays by setting values greater than 0.5 to 1 and the rest to 0. The Jaccard similarity index for each component was computed as: $J_{i}=\frac{\left|BH\cap M_{i}\right|}{\left|BH\cup M_{i}\right|}$, where $M_{i}$, i=0,1,2,3 represents the binary segmentation of component *i* (0 = background, 1 = vasculature, 2 = non-tumor nuclei, 3 = tumor nuclei), and BH denotes the binary Delta-Map in each sample. To establish a baseline for comparison, computed the Jaccard index against segmentation masks from randomly selected patches within the same sub-transition grade. Next, we computed ratio of true and random Jaccard indexes for each patch and reported the median value across all patches for a grade transition (Table in Fig. 4C). Additionally, we assessed the statistical significance of enrichment in specific cell types by comparing the Delta-map similarity against the randomized baseline, using a one-sided T-test (Bar plot in Fig. 4C).

### Nucleoli Detection

Since standardized nucleolus detection algorithms are unavailable, we developed a heuristic approach that identifies “nucleoli” as intensity peaks in Hematoxylin-stained sections of H&E images. We first convert the image to the HED color space and isolate the hematoxylin channel, normalizing and smoothing it using Gaussian distribution with standard deviation δ=1 to enhance nucleoli detection. We then identify nuclei on the nuclei mask, distinguishing between non-tumor and tumor nuclei based on our pretrained nuclei segmentation. “Nucleoli” are detected as local maxima (prominence threshold α=0.08) in the smoothed hematoxylin image, representing darker, separate spots within the nuclei. However, as nuclei themselves represent local peaks in hematoxylin intensity, this approach initially detected “nucleoli” in nearly all nuclei (Fig. S9). To correct this, we subtract one nucleolus from each nucleus with detected nucleoli. Finally, we calculate the average number of “nucleoli” per nucleus for each type, providing quantitative nucleoli statistics.

### Counterfactual analysis on real and synthetic data

To highlight Delta-March’s ability to improve statistical power in detecting underlying biological mechanisms, we performed a counterfactual analysis (Fig. 4F) examining average nuclear area across tumor grades 1 to 4. We analyzed latent traversal from grade 1 to grade 4 with 977 corresponding patches per grade. For real data, we sampled 977 patches per grade to maintain comparability with synthetic data. Samples for each grade-pair (N = 100 patches per group) are obtained through three distinct strategies: (1) Real-Unpair: Randomly sampled image patches from each grade group using the real dataset, (2) Syn-Pair: Synthetic image patches for each grade pair derived from the same random latent trajectory, thus preserving progression continuity in the synthetic generation, (3) Syn-Unpair: Synthetic image patches from independent latent trajectories, simulating samples from unrelated transitions. To quantify differences in nuclei, we applied the sign test to compare the per-patch average nuclear area between the two grades. The entire process was repeated 1,000 times to assess the robustness and consistency of the statistical signal, measured by the mean log-transformed P-value.

## Supplementary Material

Supplement 1

## Data Availability

WSI for the Mayo cohort are available at https://doi.org/10.25452/figshare.plus.19310870. All code used in this paper, including a Jupiter notebook to generate all figures, is attached as supplementary material and will be released on the Rajaram Lab GitHub page upon acceptance.
