# Supplementary material for: Autonomous learning of pathologists’ cancer grading rules
Source: bioRxiv. 2025 Mar 19:2025.03.18.643999. Preprint. [Version 1] doi: 10.1101/2025.03.18.643999 (PMC11956981; doi:10.1101/2025.03.18.643999)
Supplement: Supplementary file 1 [file media-1.pdf]

Supplementary figures

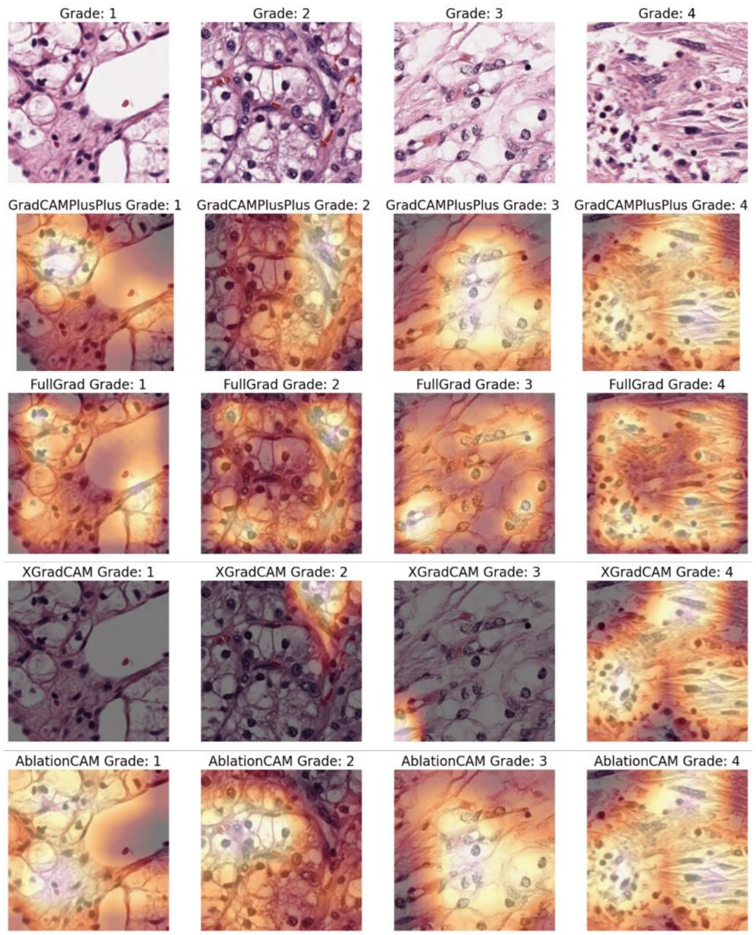

Figure S1: Class Activation Map (CAM)-based heatmaps for tumor grade classification exhibit a diffuse distribution, lacking precise localization and clear interpretability of the critical features relevant to tumor grading. This figure presents representative CAM-heatmaps for our pretrained VGG19-based grade classifier on images of varying grades (columns) based on different CAM approaches (rows: GradCam++, FullGrad, XGradCAM, and AblationCAM). Heatmaps visualize activations on the last convolution layer in predicting the correct grade class.

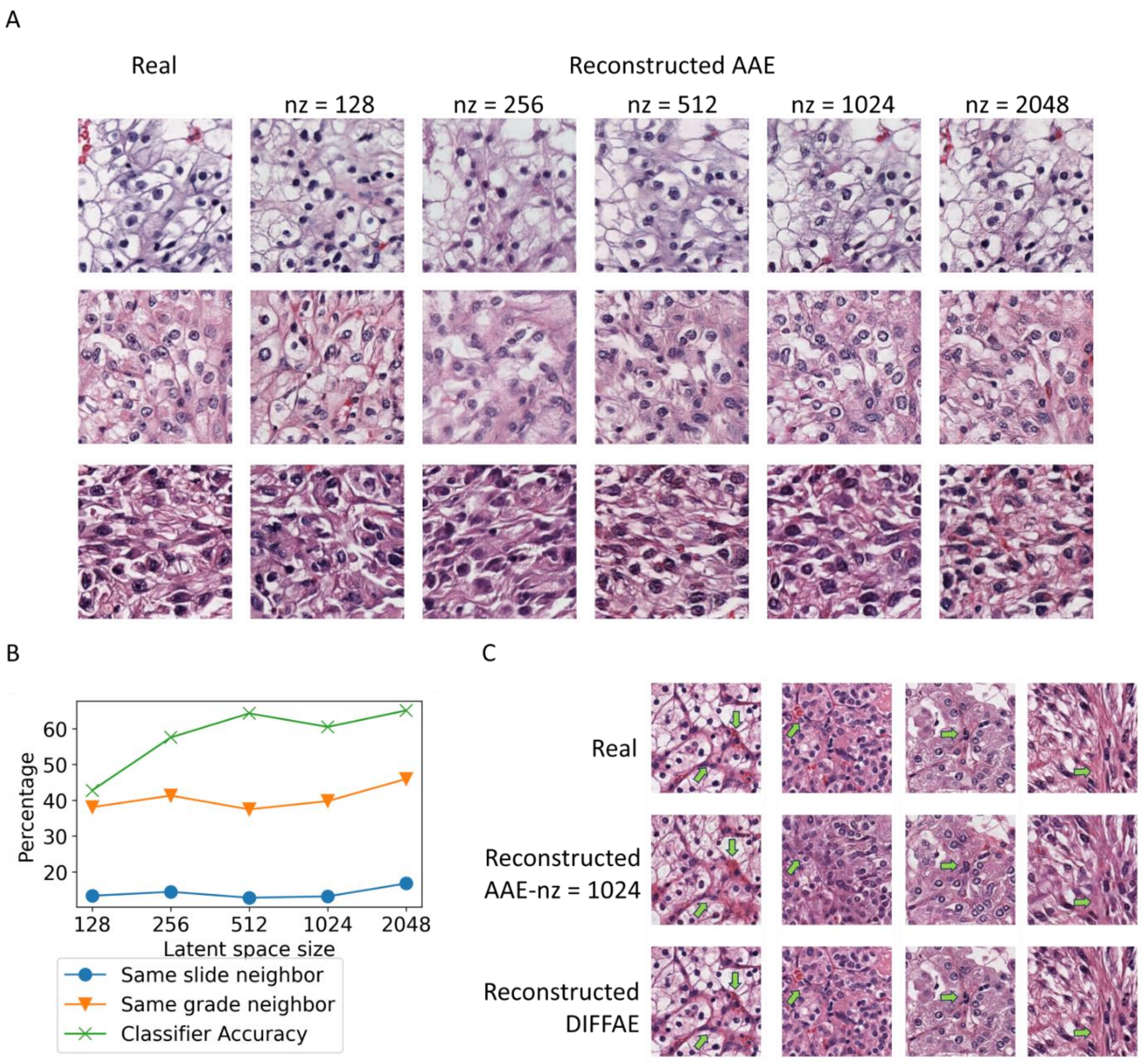

Figure S2: Performance of the Adversarial Autoencoder (AAE) in reconstructing histopathological images. (A) Examples of 3 images (rows) reconstructed using the AAE model with varying latent space dimensions (columns, nZ = 128, 256, 512, 1024, 2048). **(B)** Effect of AAE latent space dimensionality on latent space quality as assessed by different metrics. Blue/Orange plots: average percentages of neighboring patches of a patch having the same slide/grade respectively. Green plots: LDA classifier accuracy in each latent space (N=120,000 patches) **(C)** Additional examples (beyond Fig. 2F) of reconstructed images obtained by AAE model with 1024-dimensional latent space and DIFFAE model. While quality is high in both autoencoders, there are several subtle but biologically significant differences highlighted by arrows.

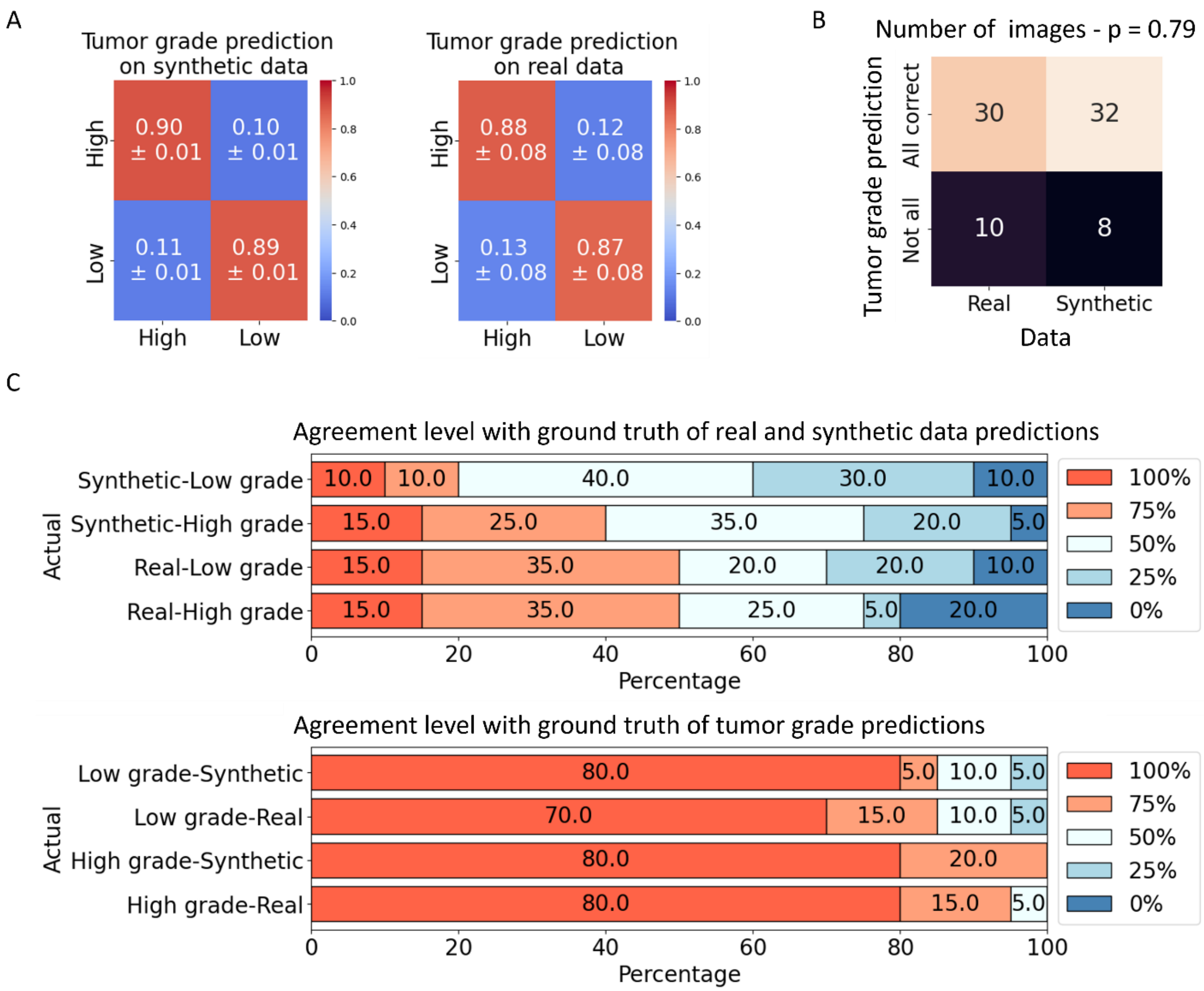

Figure S3: Synthetic data captures grading nuances, enhancing pathologists' tumor grade prediction. We conducted a survey using 80 images, comprising 40 real pathological images and 40 synthetic images generated by DIFFAE models. Each group (real and synthetic) includes 10 patches per grade. Pathologists were tasked with assessing whether the image was (i) real or synthetic and (ii) low (Grade 1, 2) or high (Grade 3, 4) grade. **(A)** Mean and standard deviation of tumor grade accuracy on synthetic (left) and real (right) data, with ground truth based on pathologist-assigned grades. **(B)** Matrix depicting the distribution of correct tumor grade calls by all pathologists in real vs synthetic data (N=40). P-value (shown in the title) is computed using Fisher's exact test to assess differences between the results of the two data groups. **(C)** The images (N=80) were categorized into different agreement levels (100%, 75%, 50%, 25%, 0%) based on the percentage of pathologists who agreed on whether the image was real or synthetic (Top) and whether the tumor grade was high or low (Bottom). Ground truth in (B, C) is obtained from tumor grade classification model.

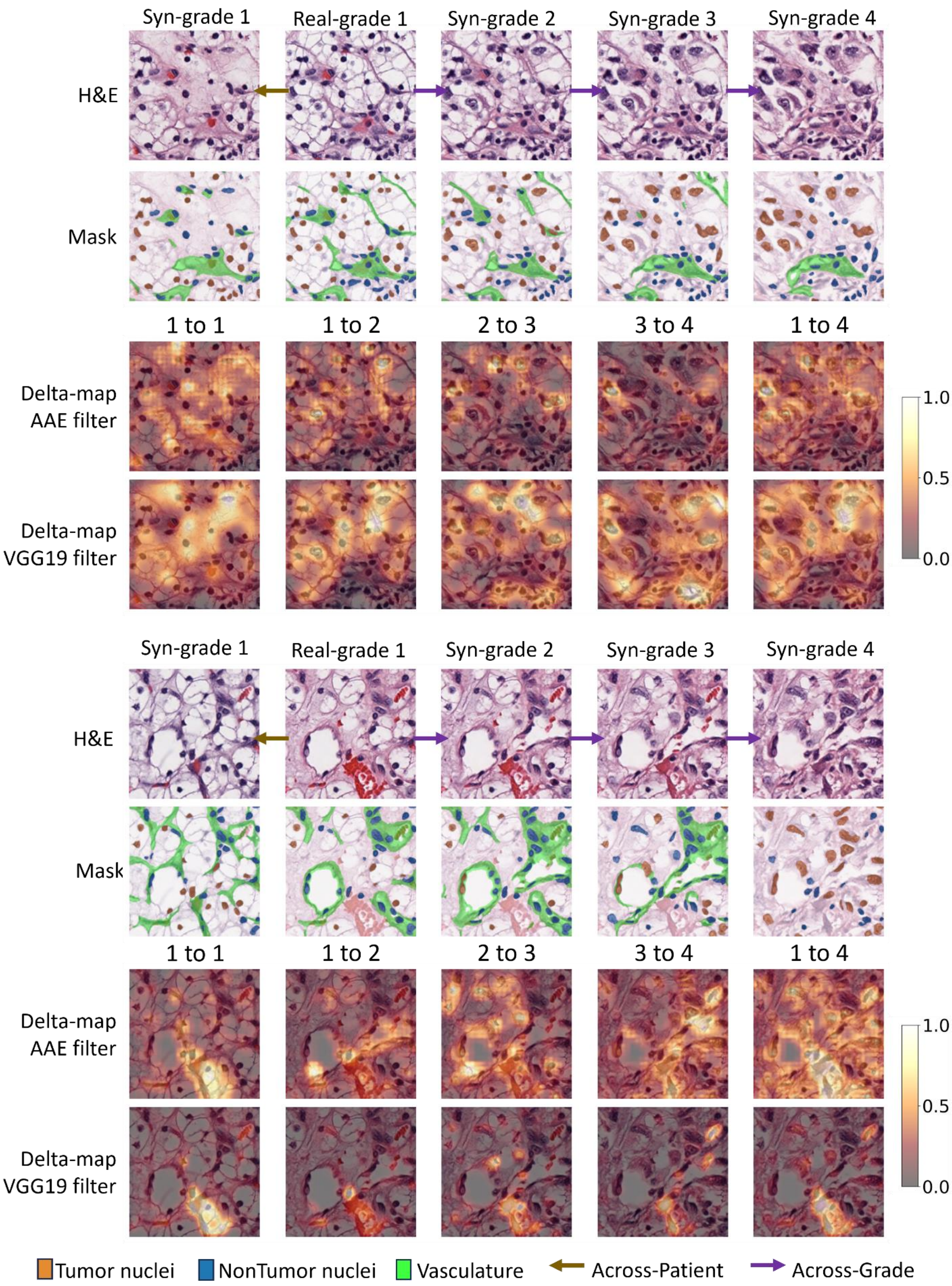

Syn-grade 1
Real-grade 1
Syn-grade 2
Syn-grade 3
Syn-grade 4
H&E
Mask
1 to 1
1 to 2
2 to 3
3 to 4
1 to 4
Delta-map AAE filter
Delta-map VGG19 filter
1.0
0.5
0.0
Syn-grade 1
Real-grade 1
Syn-grade 2
Syn-grade 3
Syn-grade 4
H&E
Mask
1 to 1
1 to 2
2 to 3
3 to 4
1 to 4
Delta-map AAE filter
Delta-map VGG19 filter
1.0
0.5
0.0
Tumor nuclei
NonTumor nuclei
Vasculature
Across-Patient
Across-Grade

Fig S4: Additional examples (beyond Fig. 4B) of across-patient and across-grade transitions. Analysis was performed as in Fig 4B, except we show the activation using a grade classification model in addition to the AEE. In the across-patient (grade 1 to grade 1) transition the resulting synthetic tissues maintain the overall spatial architecture of nuclei while exhibiting distinct tissue styles in grade 1. This underscores DIFFAE's ability to disentangle tissue-specific features and generate semantically meaningful representations. In the across-grade transition, real tissue semantic vectors are incrementally shifted toward grade 4. Intermediate points along this semantic trajectory are captured and synthesized with the original stochastic terms, generating synthetic tissues corresponding to grades 2, 3, and 4. Delta maps were computed using CNN filters from the first 9 layers of the pretrained AAE and the first 13 layers of the VGG19 classifier.

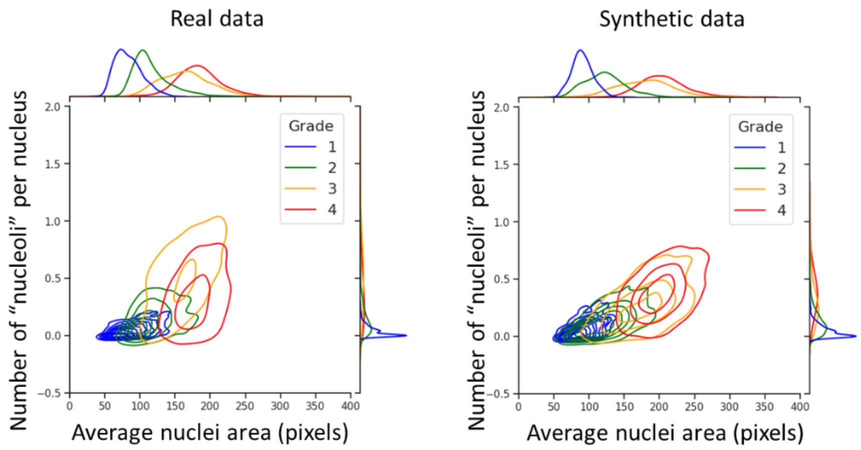

Figure S5: Synthetic images show greater consistency between different grade associated features than real images. Contour plots show the correlation between tumor nuclei size and "nucleoli" number in real data and in synthetic data (N=977 patches per grade).

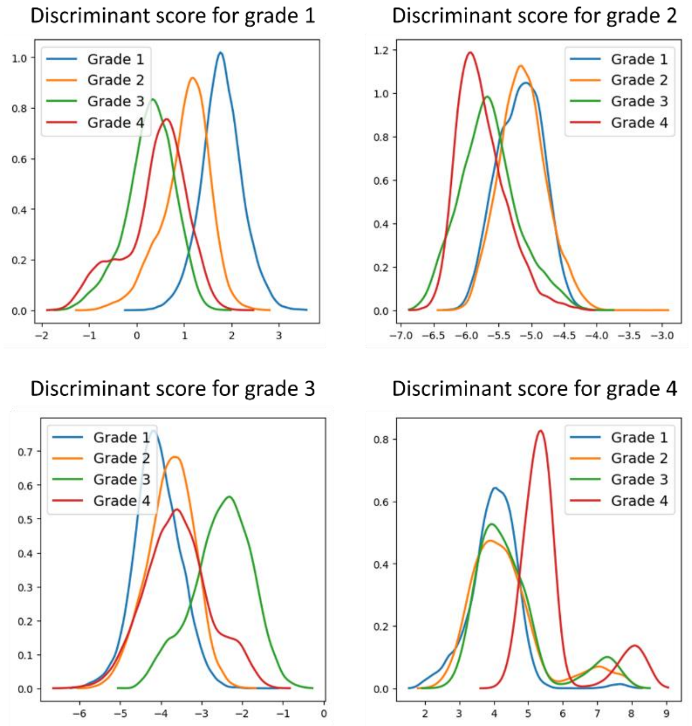

Figure S6: Projection of latent space onto classifier weight vectors for grade transitions. The figure shows projections of the latent space onto the weight vectors of a linear classifier trained on latent vectors (N=8,000). Each panel illustrates the discriminant score distributions for grades 1 through 4. The grade 4 vector shows a continuous progression from grade 1 to grade 4, while the grade 1 vector displays an inverse trend.

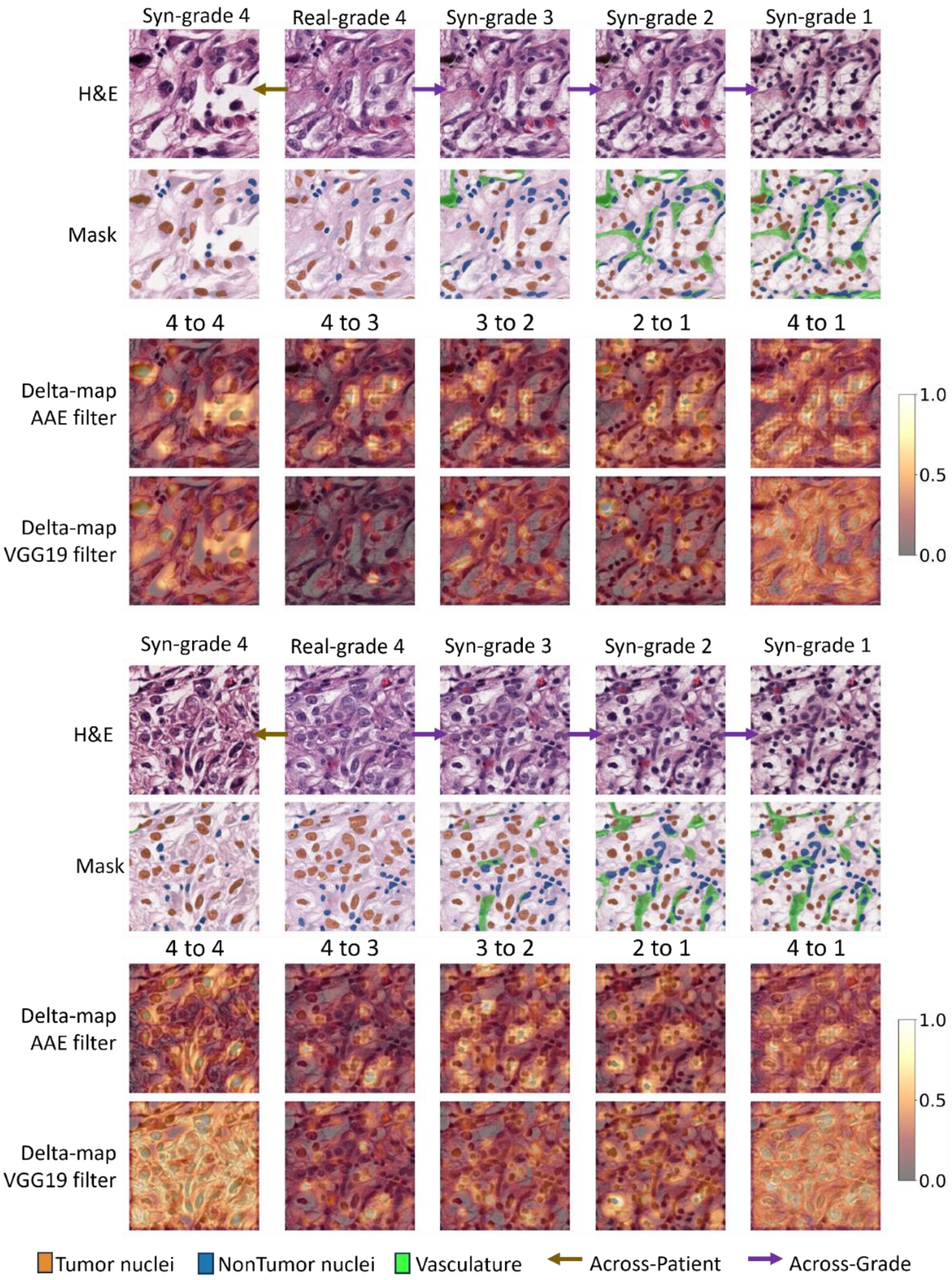

Fig S7: Examples of across-patient and across-grade transitions starting from grade 4 with corresponding segmentation masks and Delta marches from CNN layers of the AAE model and VGG19 classifier. This figure shows two latent transitions starting from grade 4 with similar analysis performed in Fig. S4 (and Fig. 4B). Real grade 4 tissue images from different patients were processed by DIFFAE, extracting their semantic vectors and stochastic terms. For the across-

grade transition, grade 4 semantic vectors were gradually shifted toward grade 1, generating synthetic tissues for grades 3, 2, and 1. Delta march heatmaps capture significant morphological changes in nuclei across grades. Additionally, differences in nuclei texture between real and synthetic tissues within grade 4 are evident.

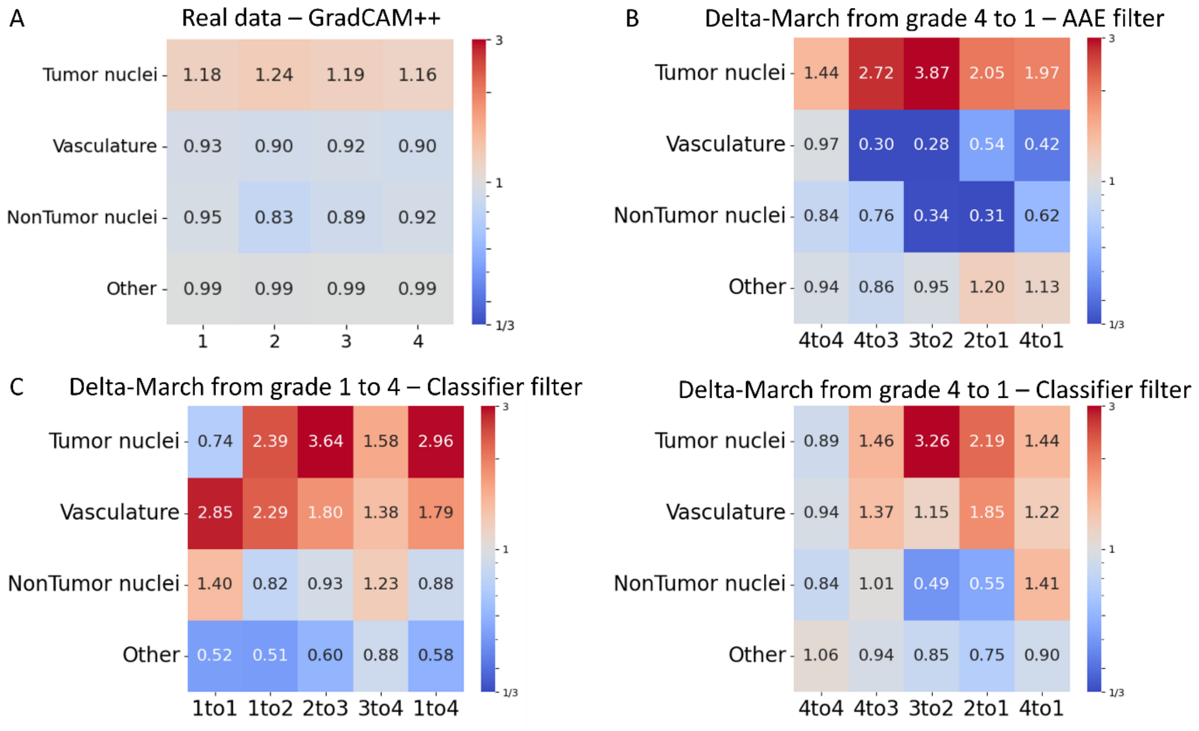

Fig S8: Effect of IDL strategy and filter model on cell type localization. We evaluate various filter models to generate activations and report the median Jaccard similarity ratio between actual and random masks. The binarization of activations and computation of the Jaccard ratio follow the same procedure as in Fig. 4C. **(A)** The median Jaccard ratio is calculated using activations obtained from Grad-CAM++ applied to real data, with 1,000 patches per grade. The random mask for each sample is taken from the other 999 masks having the same grade. **(B)** and **(C)** present the Jaccard ratios for activations (Delta-maps) generated by the Delta-March approach using different CNN filters: **(B)** the first 9 layers of an AAE model (beyond Fig. 4C), and **(C)** the first 13 layers of a VGG19-based grade classification model. We analyze two latent traversals: one transitioning from grade 1 to grade 4 (N=977 samples) and another from grade 4 to grade 1 (N=557 samples), each sample reaches all four grades during transition.

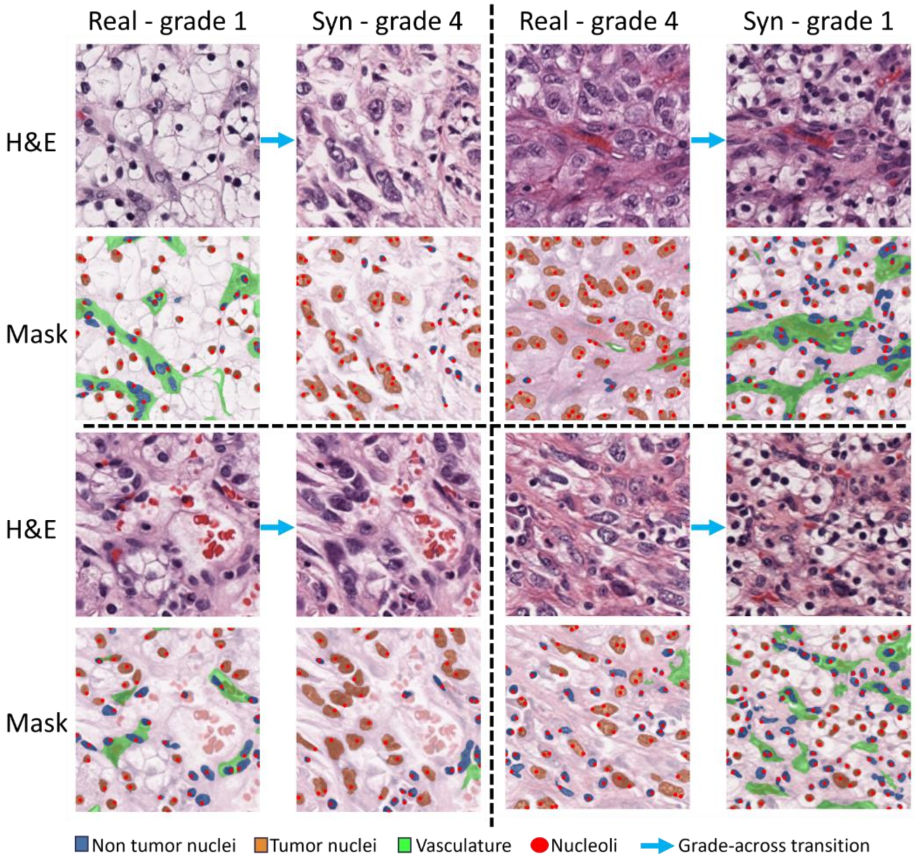

Figure S9: Nucleoli detection in Hematoxylin sections using image processing techniques. The figure presents examples of nucleoli detection within nuclei from Hematoxylin sections, leveraging the HED color space and isolating the hematoxylin channel. Nucleoli, identified as darker, separate spots, are detected as local maxima within smoothened images. Despite occasional false nucleoli predictions in non-tumor nuclei due to resolution limitations, the method reveals a higher average number of nucleoli in synthetic high-grade images compared to lower-grade images.
